# Atlas of Skin Diseases

**Published:** 1879-05

**Authors:** 


Atlas of Skin Diseases. By Louis A. Duhring, M.D., Professor of Skin Diseases in the Hospital of the University of Pennsylvania. Part V. J. B. Lippincott & Co., Philadelphia. 1879.

This is another issue of this valuable contribution to the practical and clinical study of skin diseases. It is not necessary to reiterate our high opinion of this enterprise. Part fifth contains chromo-photographs of scabies, herpes zoster, tinea sycoses and eczena (vesiculosus), with explanatory remarks. Practical demonstration and information may be derived from this work, almost equal to any nosocomial course of study that might be instituted.

